# Comparative Metagenomics Reveals the Distinctive Adaptive Features of the *Spongia officinalis* Endosymbiotic Consortium

**DOI:** 10.3389/fmicb.2017.02499

**Published:** 2017-12-14

**Authors:** Elham Karimi, Miguel Ramos, Jorge M. S. Gonçalves, Joana R. Xavier, Margarida P. Reis, Rodrigo Costa

**Affiliations:** ^1^Microbial Ecology and Evolution Research Group, Centre of Marine Sciences, University of Algarve, Faro, Portugal; ^2^Fisheries, Biodiversity and Conservation Research Group, Centre of Marine Sciences, University of Algarve, Faro, Portugal; ^3^Department of Biology and K.G. Jebsen Centre for Deep Sea Research, University of Bergen, Bergen, Norway; ^4^Faculty of Science and Technology, University of Algarve, Faro, Portugal; ^5^Institute for Bioengineering and Biosciences, Department of Bioengineering, Instituto Superior Técnico, Universidade de Lisboa, Lisbon, Portugal

**Keywords:** host–microbe interactions, marine sponges, microbiome, next-generation sequencing, symbiosis

## Abstract

Current knowledge of sponge microbiome functioning derives mostly from comparative analyses with bacterioplankton communities. We employed a metagenomics-centered approach to unveil the distinct features of the *Spongia officinalis* endosymbiotic consortium in the context of its two primary environmental vicinities. Microbial metagenomic DNA samples (*n* = 10) from sponges, seawater, and sediments were subjected to Hiseq Illumina sequencing (*c.* 15 million 100 bp reads per sample). Totals of 10,272 InterPro (IPR) predicted protein entries and 784 rRNA gene operational taxonomic units (OTUs, 97% cut-off) were uncovered from all metagenomes. Despite the large divergence in microbial community assembly between the surveyed biotopes, the *S. officinalis* symbiotic community shared slightly greater similarity (*p* < 0.05), in terms of both taxonomy and function, to sediment than to seawater communities. The vast majority of the dominant *S. officinalis* symbionts (i.e., OTUs), representing several, so-far uncultivable lineages in diverse bacterial phyla, displayed higher residual abundances in sediments than in seawater. CRISPR-Cas proteins and restriction endonucleases presented much higher frequencies (accompanied by lower viral abundances) in sponges than in the environment. However, several genomic features sharply enriched in the sponge specimens, including eukaryotic-like repeat motifs (ankyrins, tetratricopeptides, WD-40, and leucine-rich repeats), and genes encoding for plasmids, sulfatases, polyketide synthases, type IV secretion proteins, and terpene/terpenoid synthases presented, to varying degrees, higher frequencies in sediments than in seawater. In contrast, much higher abundances of motility and chemotaxis genes were found in sediments and seawater than in sponges. Higher cell and surface densities, sponge cell shedding and particle uptake, and putative chemical signaling processes favoring symbiont persistence in particulate matrices all may act as mechanisms underlying the observed degrees of taxonomic connectivity and functional convergence between sponges and sediments. The reduced frequency of motility and chemotaxis genes in the sponge microbiome reinforces the notion of a prevalent mutualistic mode of living inside the host. This study highlights the *S. officinalis* “endosymbiome” as a distinct consortium of uncultured prokaryotes displaying a likely “sit-and-wait” strategy to nutrient foraging coupled to sophisticated anti-viral defenses, unique natural product biosynthesis, nutrient utilization and detoxification capacities, and both microbe–microbe and host–microbe gene transfer amenability.

## Introduction

Sponges (phylum Porifera) rank among the oldest extant metazoans and are distributed worldwide across all oceans and major freshwater bodies, displaying various shapes, sizes, and colors, which are possibly influenced by environmental and biotic conditions (Hentschel et al., 2006; Pineda et al., 2015). There are about 8,500 sponge species described to date and likely as many to be described (Van Soest et al., 2012). These sessile, filter-feeding organisms usually shelter dense and complex microbial communities often dominated by diverse, active, and phylogenetically distinct bacteria (Taylor et al., 2007; Kamke et al., 2010; Thomas et al., 2010). Indeed, although sponges intake numerous planktonic microorganisms due to their remarkable filtering activity, their symbiotic communities are taxonomically and functionally different from those found in the water body (Thomas et al., 2010, 2016; Costa et al., 2013). Until now, little experimental evidence exists for the actual participation of sponge symbionts in contributing to host fitness (Webster and Thomas, 2016). However, sponge-associated microorganisms are believed to benefit their hosts through several, eventually interdependent, mechanisms. These include nutrient provision (e.g., through the synthesis of photosynthates and vitamins; Taylor et al., 2007; Siegl et al., 2011); *in-host* geochemical cycling (e.g., via nitrification; Bayer et al., 2008; Radax et al., 2012), denitrification (Siegl et al., 2011; Fan et al., 2012), or polyphosphate production (Zhang et al., 2015); chemical defense (e.g., via the biosynthesis of polyketides; Piel et al., 2004; Wilson et al., 2014); and removal of metabolic by-products such as ammonia (Webster and Taylor, 2012; Webster and Thomas, 2016) and sulfide (Hoffmann et al., 2005). Particularly, the phylogenetic distinctiveness of the marine sponge microbiome and its vast natural product biosynthesis repertoire have both propelled much research interest in this symbiotic relationship (Taylor et al., 2007; Wilson et al., 2014).

In the last 10 years or so, metagenomics (Handelsman, 2007) and single cell genomics (SCG) (Woyke et al., 2009) approaches coupled to next-generation sequencing (NGS) technologies have become the tools of trend in the inspection of microbial communities thriving in open and host-associated microniches (Handelsman, 2007; Kennedy et al., 2008; Gilbert and Dupont, 2011; Kumar et al., 2015). Functional gene profiling via shotgun NGS revealed that sponge symbiont communities share a suite of common genetic signatures underlying “specific” adaptive strategies such as high frequencies of eukaryotic-like proteins (ELPs), possibly involved in patterns of host–symbiont recognition, and Clustered Regularly Interspaced Short Palindromic Repeats and associated systems (CRISPR-Cas), that may function as a collective anti-viral defense system within the sponge symbiotic consortium (Thomas et al., 2010; Fan et al., 2012; Rua et al., 2015; Horn et al., 2016). However, the frequency and abundance of such genetic elements in other marine microhabitats have not yet been fully examined, making it difficult to diagnose them as exclusive adaptive features of marine sponge symbionts. The linkage between identity and function has been now identified for a number of symbiotic lineages, either via SCG or genome binning from metagenomes (Siegl et al., 2011; Moitinho-Silva et al., 2017; Slaby et al., 2017), greatly increasing our knowledge of the potential physiology of particular sponge-enriched lineages belonging, e.g., to the *Cyanobacteria*, *Proteobacteria*, and *Poribacteria* phyla (Kamke et al., 2013; Gao et al., 2014; Burgsdorf et al., 2015).

In spite of the continued progress enabled by modern cultivation-independent tools, our current understanding of marine sponge microbiome diversity and function mostly derives from comparative studies with the neighboring bacterioplankton (Fan et al., 2012; Rua et al., 2015; Thomas et al., 2016), whereas knowledge of the potential contribution of sediments as sinks and sources of sponge-associated bacteria remains limited. Only recently have studies emerged which investigated sediments in comparative analyses with sponge symbiotic assemblages, using amplicon-based approaches to address the taxonomy and, eventually, *in silico* functional estimates of the examined communities (Polónia et al., 2014; Thomas et al., 2016). Recent evidence suggests that the density and biochemical composition of particles are major drivers of microbial community structure in aquatic microniches (Zhang et al., 2016). Here, we hypothesize that higher particle/surface availability and cell densities may promote the selection of identifiable traits common to sponge-associated and sediment communities not necessarily favored in planktonic settings. To address this hypothesis, in this study, we tested whether (1) whole taxonomic and functional profiles and (2) abundance distributions of genotypic traits usually regarded as adaptive features of marine sponge symbionts were significantly different across sponge, sediments, and seawater microbial metagenomes.

*Spongia officinalis* Linnaeus 1759, the first described sponge species, is a canonical bathing sponge (Voultsiadou et al., 2008) displaying widespread occurrence from across the Mediterranean Sea (Dailianis et al., 2011) into the northeastern Atlantic Ocean and beyond. However, signs of population decline as a consequence of human activity, warming temperatures, and bacterial infections have been accumulating in recent years (Webster, 2007; Garrabou et al., 2009). *S. officinalis* belongs to the chemically rich order Dictyoceratida (Gordaliza, 2010), and as such is the source of diverse biologically active natural products (Gonzalez et al., 1984; Manzo et al., 2011). In spite of the unequivocal economic and societal relevance of *S. officinalis*, functional information concerning its symbiotic community is scarce. Here, we employ *S. officinalis* as a model organism to quantitatively address the functional and taxonomic (dis)similarity between sponge, sediment, and seawater microbiomes using shotgun metagenomic sequencing. To reveal the distinctive genomic features of the *S. officinalis* symbiotic consortium in the context of its natural environment, we used customized pipelines enabling differential abundance analysis of symbionts [i.e., operational taxonomic units (OTUs) set at 97% 16S rRNA gene similarity] and predicted protein families/domains/sites [i.e., InterPro (IPR) entries] across the studied biotopes. Alternative analytical pipelines were used to verify the consistency of the major trends found, and to compare the *S. officinalis* microbial metagenome with those of other sponge hosts.

## Materials and Methods

### Sampling and Sponge Identification

Sampling of *S. officinalis* specimens (*c.* 10 g, *n* = 4), seawater (2 L, *n* = 3) and sediments (*c.* 50 g of upper 5 cm layer, *n* = 3) took place in May 2014 by SCUBA diving at 20 m depth off the coast of Pedra da Greta (36° 58′ 47.2N;7° 59′ 20.8W), Algarve, southern Portugal. Seawater samples were taken 1 m above the sponge specimens, while sediment samples were taken 1 m away from the sampled specimens. Underwater procedures and sample transportation were as described previously (Hardoim et al., 2012). Water pH was 8.13, temperature 18°C, and salinity 36.40aaa. Sponge individuals were identified in the laboratory using standard macro- and microscopic morphological criteria (Hardoim et al., 2012). To aid the traditional identification of the specimens, phylogenetic inference of the subunit I of the mitochondrial cytochrome oxidase (CO1) gene was undertaken. To this end, total community DNA (TC-DNA) was directly extracted from 0.25 g of the inner body of each specimen (see below). Amplification, sequencing, and phylogeny of CO1 genes were performed using previously established procedures (Hardoim et al., 2012; Hardoim and Costa, 2014).

### Microbial Metagenomic DNA Extraction and NGS

For the analysis of the sponge-associated endosymbiotic community, microbial cell pellets were retrieved from 2.5 g of the inner sponge body as detailed elsewhere (Hardoim et al., 2014). Briefly, cell homogenates obtained from the samples by maceration in calcium/magnesium free artificial seawater (CMFASW; Garson et al., 1998) were subjected to a differential centrifugation step adapted from earlier protocols (Fieseler et al., 2006; Thomas et al., 2010). Seawater samples (2 L) were passed through 0.22 μM nitrocellulose membranes which were thereafter cut into small pieces, whereas 0.25 g of sediment were retrieved from each sample after aseptic sieving (1 mm mesh) and thorough homogenization. All processed samples, including excised sponge pieces used for phylogenetic inference (see above), were stored at -80°C prior to TC-DNA extraction with the UltraClean^®^ Soil DNA isolation kit (MO BIO, Carlsbad, CA, United States) following the manufacturer’s instructions. TC-DNA quantity and concentration were determined using the Qubit (Life Technologies Qubit 2.0^®^) dsDNA HS Assay Kit. Next generation TC-DNA sequencing was performed on an Illumina Hiseq 2500 device at Mr. DNA (Shallowater, TX, United States). DNA libraries were prepared for sequencing using the Nextera DNA Sample preparation kit (Illumina) after the manufacturer’s instructions, and sequenced paired end for 200 cycles with sequence depth calibrated at *c.* 15 million 101-bp reads per sample.

### Metagenome Data Processing

Preliminary data processing and analysis revealed that, for most highly ranked taxa (domains, phyla, classes), no sensible changes in microbial community composition could be detected between assembled and unassembled data. However, assembly procedures often reduced considerably the total number of reads that could be used in downstream analysis, especially for sediment samples (Supplementary Appendix S1). Therefore, for the purposes of this study, we primarily employed complementary tools within the Meta-Genome Rapid Annotation using Subsystems Technology server (MG-RAST) v3.0 (Meyer et al., 2008) and the EBI Metagenomics (EMG) platform v2.0 (Mitchell et al., 2016) to deliver accurate metagenomic profiling from unassembled reads, making optimal use of all information generated by our sequencing effort. Prediction of coding sequences (cds), translation into protein sequences and annotation searches were performed using default settings in both MG-RAST and EMG (hard-coded data processing). Briefly, within MG-RAST gene calling was performed using FragGeneScan (Rho et al., 2010), and predicted cds were translated into proteins with clustering at 90% identity level using uclust (Edgar, 2010). Within the EMG pipeline, after quality filtering and length trimming, reads with rRNA sequences were detected using RNA Selector and subjected to taxonomic profiling using QIIME for OTU picking, clustering (at 97% gene similarity) and classification. Reads with rRNA masked were subjected to cd prediction using FragGeneScan, and predicted cds were finally processed with InterProScan for functional annotation against the IPR database release 31.0^1^, which integrates several protein sequence databases such as Pfam, TIGRFams, and PANTHER, among others.

With MG-RAST we extracted an “all domains-all reads” profile of microbiome structure based on all sequences (including “phylogenetic marker” and “functional” genes) that could be assigned a taxonomic origin. Sequencing reads were annotated using the best-hit annotation tool against the M5NR database (Wilke et al., 2012). The stringency of the BLAST parameter was a maximum e-value of 1e^-5^, a minimum identity of 60%, and a minimum alignment length of 15 measured in aa for predicted proteins and in bp for RNA databases. A negligible amount (0.02%) of the reads obtained from *S. officinalis* specimens was assigned as of poriferan origin using MG-RAST, corroborating the efficiency of the microbial enrichment protocol used to process these samples. With the EMG data processing pipeline, we obtained taxonomic and functional profiles of the metagenomes based on 16S rRNA genes (archaeal, bacterial, and microeukaryotic—chloroplast and mitochondrial—OTUs) and IPR protein domain entries, respectively, fetched from the data (Mitchell et al., 2016). Our downstream statistical analyses focused primarily on the OTU and IPR contingency tables delivered using the EMG pipeline given the high dominance of bacterial reads (>95% of the classifiable reads) verified using MG-RAST, and the possibility to explore the widely integrative, comprehensive and updated IPR protein sequence database (Finn et al., 2017). Complementary analyses on COG annotations derived from both unassembled and assembled data were performed, and are detailed below.

### Metagenome Data Analysis

The contingency (OTU and IPR) tables extracted from the EMG data processing pipeline were imported into R version 3.2.4 (R Development Core Team, 2012) using the read.delim() function. Since differences in library sizes among samples did not require rarefaction of the data to the least sequenced samples (McMurdie and Holmes, 2014; Weiss et al., 2017) analyses were performed on the full OTU and IPR datasets after Hellinger transformation of the data. This procedure was found to perform better than using relative abundances alone to assess variability in IPR and OTU data across samples by preventing the emergence of false positives and down-weighting the impact of very dominant IPR entries (usually representing primary metabolic traits) on the determination of most differentiating functional attributes among biotopes. Variation in taxonomic (OTU) and functional (IPR) microbial community structures across sediment, seawater and sponge samples was assessed by principal coordinates analysis (PCoA) using Bray–Curtis dissimilarity matrices as input data within the cmdscale() function in R. Differences were tested for significance by permutational analysis of variance (PERMANOVA) using the above-mentioned matrices and the adonis() function within the VEGAN package, with the number of permutations set at 1000. Similarity Percentage (SIMPER) (Clarke, 1993) analyses were performed with the PAST software v. 3.14 (Hammer et al., 2001) to rank the individual contribution of each annotated OTU and IPR to total data variation in taxonomic and functional profiles, respectively. Analyses performed using Euclidean (instead of Bray–Curtis) distances led to equivalent outcomes and are available on request.

Pairwise tests of significance were run to diagnose differences in IPR, OTU, and phylum relative abundances among biotopes with the sim() function in R using Hellinger-normalized data as input. Heat maps were generated to display the top, most differentiating microbial phyla, OTUs, and IPR entries (identified via SIMPER analyses) using the heatmap2() function in the gplots package within R. Additionally, we manually inspected all IPR entries oscillating significantly among the biotopes to identify potential “umbrella” functions of likely ecological and evolutionary relevance for sponge microbiome assembly, including traits usually regarded as “specific” genomic signatures of sponge symbionts, and assessed the cumulative contribution of all IPR entries belonging to these so-created, major functional categories in distinguishing between the biotopes. To test whether abundance values of major functional categories assembled manually varied significantly among the biotopes, the Shapiro–Wilk statistics was computed in R to inspect the distribution of each measure around means. Thereafter, one-way ANOVA was performed followed by an all pairwise multiple comparison procedure using the Tukey’s HSD (honest significant difference) test. For non-normal distributed data, Kruskal–Wallis one-way ANOVA by ranks was employed followed by *post hoc* Kruskal–Nemenyi tests for pairwise multiple comparisons. The same strategy was applied to test for differences among Bray–Curtis dissimilarities between samples, calculated for both IPR and OTU data.

### Alternative Analytical Pipelines and Data Validation

Besides the core analyses described above using EMG taxonomic and functional profiling of unassembled reads, we performed COG-based annotations of both unassembled and assembled reads on MG-RAST. Assembly of metagenomes was carried out using MetaVelvet (Namiki et al., 2012) with default parameters. Thereafter, assembled and unassembled data were processed within MG-RAST as described above. Predicted protein sequences were searched against the COG database (Tatusov et al., 2003) using a maximum e-value of 1e^-10^, minimum identity of 60%, and minimum alignment length of 15 aa. The resulting COG vs. samples tables, for unassembled and assembled data, were then subjected to ordination analysis using Hellinger transformation and PCoA, as described above, to test whether COG profiles were different according to their origin (i.e., *S. officinalis*, seawater, and sediments). To contrast the functional profiles retrieved from *S. officinalis* with those obtained for other sponge hosts, we downloaded the COG annotations available on MG-RAST (study:Bbay metagenome mgp5369) describing the microbiomes of *Rhopaloeides odorabile* (id: mgm4530290.3), *Cymbastela concentrica* (id: mgm4530252.3), and *Cymbastela coralliophila* (id: mgm4530427.3) (Fan et al., 2012) and merged them with COG annotations retrieved in this study in a single file. Only assembled data were used in this comparison. The resulting COG vs. samples matrix was subjected to ordination analysis after Hellinger data transformation as delineated above, however, here ordination was carried out using both the full and rarefied (number of annotated reads per sample standardized to the sample with the lowest number of reads) COG datasets. Venn diagrams were constructed using Venny 2.1.0^2^ to count the number of specific and shared COGs across the four analyzed sponge species. Finally, the COGs assigned to the microbiomes of all four sponge species were lumped together and subjected to SIMPER analysis against sediment and seawater metagenomes to rank COG entries contributing the most to differentiate between sponge (all species), sediment, and seawater biotopes. All results deriving from these analyses are described in detail as Supplementary Material (Supplementary Appendix S1).

### Nucleotide Sequence Accession Numbers

Sponge CO1 sequences were deposited in the National Center for Biotechnology Information (NCBI^3^) under the accession numbers KX574847 to KX574851. All metagenomes are accessible through the MG-RAST (project ID 13419_021215RCmetagenomes) and EMG platforms (project # ERP012972), and were deposited in the European Nucleotide Archive (ENA^4^) under the accession numbers ERR1103453 to ERR1103462.

## Results

### Sponge Identification

Sponge specimens were identified as *S. officinalis* (Linnaeus, 1759) based on macro- and microscopic morphology coupled with phylogenetic inference of the CO1 gene. Analysis of CO1 diversity (Supplementary Figure S1) revealed 100% homology between the nucleotide sequences of our specimens and the Mediterranean (“MEDIT”) *S. officinalis* haplotype (GenBank accession no. HQ830362) as defined elsewhere (Dailianis et al., 2011).

### Microbial Metagenomes—Dataset Overview

About 15 million paired-end reads (including forward and reverse reads) of 100 nucleotides in length were generated per sample, totaling 15.25 Gb of sequencing information (Supplementary Table S1). Quality filtering and length trimming of reads using the EMG pipeline resulted in 103,104,001 high-quality reads (averaging 10,310,400 reads per sample) effectively used in downstream analyses (Supplementary Table S1). Overall, 20–22% of the reads per sample could be assigned a function (i.e., IPR entry) after ORF prediction and annotation with EMG, resulting in 22,156,186 annotated cds across the data, which constituted the functional analytical dataset. The number of annotated cds per sample ranged from 1,808,840 to 2,446,913 reads (Supplementary Table S1) totaling 10,272 IPR domains detected. The taxonomic analytical dataset consisted of 53,551 prokaryotic 16S rRNA gene reads identified from the data using the RNA Selector tool coupled to QIIME-driven OTUs picking and taxonomic assignment. 16S rRNA gene reads were assigned to 784 OTUs in total. Details pertaining to COG annotations performed with MG-RAST can be found in Supplementary Appendix S1.

### Functional and Taxonomic Ordination

**FIGURE 1:**
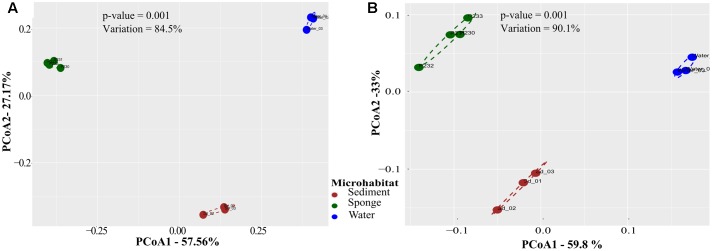
Principal coordinates analysis (PCoA) of taxonomic **(A)** and functional **(B)** microbial community profiles across biotopes. Community ordinations were based on pairwise Bray–Curtis dissimilarities (**Table 1**) calculated from normalized data, considering oscillations of relative OTU and IPR abundances among samples. Analyses were performed on OTU and IPR community profiles extracted from the 10 metagenomes using the EBI metagenomics (EMG) pipeline. Values on axes denote the extent of variation explained by each principal coordinate, whereas the total variation explained in the ordination space is indicated in the inlet. Significance values result from permutational analysis of variance (PERMANOVA) applied to the corresponding dissimilarity matrices.

PCoA performed on Bray–Curtis dissimilarity matrices calculated from normalized data revealed that sediments, seawater, and *S. officinalis* harbor highly divergent microbial communities at the finest functional (IPR entries) and taxonomic (16S rRNA gene OTUs) levels of resolution (**Figure 1**). Sponge and seawater microbial communities presented the highest levels of divergence at both the functional and taxonomic levels, whereas sponge and sediment microbiomes shared the highest extent of functional (IPR) equivalence (**Table 1**). Between-biotope community distances were significantly higher than within-biotope distances in all possible combinations (**Table 1**) corroborating the consistent trends obtained by ordination analysis (**Figure 1**). Highly divergent functional profiles from sediment, seawater, and *S. officinalis* microbiomes could as well be depicted using COG annotations of assembled and unassembled data (Supplementary Appendix S1). However, the significantly closer similarity between sponges and sediments observed with IPR functional profiling (**Table 1**) could not be re-verified employing COG annotations with MG-RAST (see Supplementary Appendix S1 for details).

**Table 1:** Functional (IPR) and taxonomic (OTU) community dissimilarities calculated between- and within-biotope samples.

Between	Sponge vs. seawater	Sponge vs. sediment	Sediment vs. seawater
IPRs	0.280 ± 0.020^a^	0.222 ± 0.016^b^	0.252 ± 0.028^c^
OTUs	0.827 ± 0.015^a^	0.718 ± 0.017^b^	0.664 ± 0.030^b^
**Within**	**Sponge**	**Seawater**	**Sediment**
IPRs	0.091 ± 0.027^a^	0.043 ± 0.002^b^	0.076 ± 0.031^ab^
OTUs	0.320 ± 0.027^a^	0.196 ± 0.017^b^	0.326 ± 0.047^a^

*Shown are average Bray–Curtis dissimilarity values ± standard deviations. Within each row, values tagged with different letters are significantly different (p < 0.05) according to one-way ANOVA, except for the OTU-based comparison between biotopes where non-parametric ANOVA on ranks was used*.

### 16S rRNA Gene Taxonomic Profiling

**FIGURE 2:**
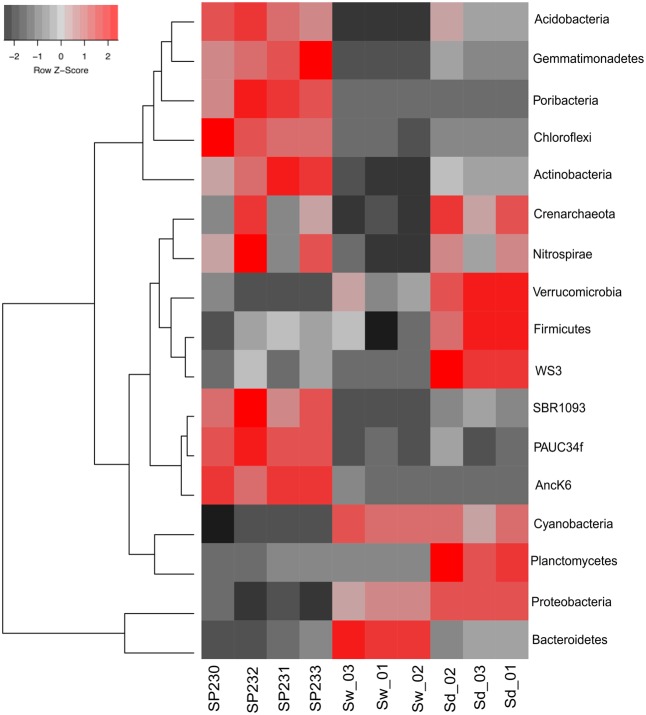
Heat map of the most differentiating microbial phyla across biotopes based on OTU data. Shown are the 17 phyla whose (OTU) relative abundances were found to oscillate the most among biotopes, explaining >85% of the variation in phylum distributions. The dendrogram clusters phylum entries according to their abundance distributions across biotopes, labeled at the bottom of the diagram. Red squares show higher relative abundance values than the mean, whereas gray squares show lower relative abundance values than the mean. Within each phylum, color intensities are determined as a linear function of the *Z*-score calculated for each phylum abundance value as the subtraction of that value by the mean divided by the standard deviation around that mean [*Z* = (*x* – mean)/SD]. SP230–P233, sponge microbial metagenomes; Sd, sediment metagenomes; Sw, seawater metagenomes.

OTUs established at 97% 16S rRNA gene similarity were fetched with the EMG pipeline (see section “Materials and Methods” for details) and used in taxonomic profiling. The *S. officinalis* symbiotic consortium was characterized by a relatively even distribution of diverse and dominant bacterial phyla, namely *Proteobacteria*, *Bacteroidetes*, *Poribacteria*, *Chloroflexi*, *Actinobacteria*, *Acidobacteria*, and *Gemmatimonadetes*, with 33 prokaryotic phyla (one archaeal, 32 bacterial) being detected across all sponge individuals (Supplementary Table S2A). In contrast, *Proteobacteria* and *Bacteroidetes* dominated the seawater microbiome, followed by *Cyanobacteria* and phytoplankton. The former were also the most abundant phyla in sediments, along with an enormous variety of less abundant groups among which *Planctomycetes*, *Crenarchaeota*, *Actinobacteria*, *Acidobacteria*, and *Verrucomicrobia* prevailed. All above-mentioned phyla significantly contributed to data variation among the three inspected biotopes (**Figure 2**) (*p* < 0.05, Supplementary Table S3A).

**FIGURE 3:**
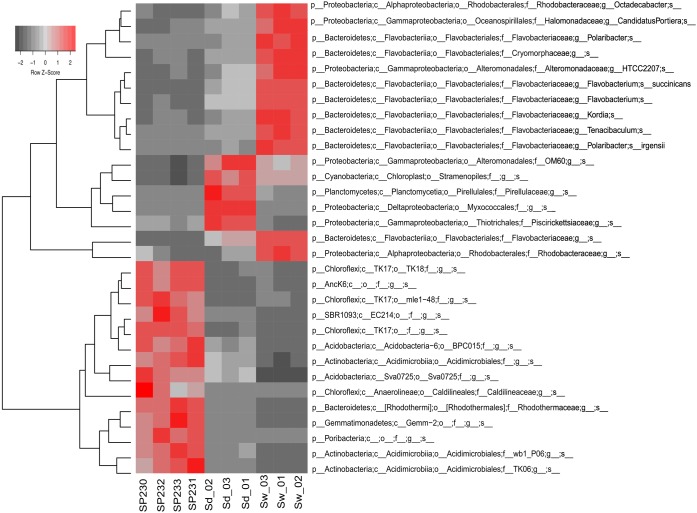
Heat map of the most differentiating OTUs across biotopes. Shown are the 31 OTUs (97% cut-off) found to oscillate the most among biotopes, explaining >32% of the variation in the OTU dataset. Heat map details are as in legend to **Figure 2**.

We detected 293, 607, and 341 16S rRNA gene OTUs in sponges, sediments, and seawater, respectively (Supplementary Table S2B). Corresponding to 63.8% of all 16S rRNA gene reads from sponges, the 10 most abundant OTUs from *S. officinalis* were, without exception, remarkably enriched in the sponge host, showing much lower abundances in the environmental vicinities (Supplementary Table S3). Noteworthy in this regard was OTU 399 belonging to the canonical sponge-enriched phylum *Poribacteria*. It dominated the *S. officinalis* microbiome accounting for 11% of all 16S rRNA genes retrieved from this source, ranking as the second OTU contributing the most to the total phylogenetic divergence computed in the taxonomic dataset (Supplementary Table S3B). The 25 most dominant *S. officinalis* OTUs comprised 86.9% of all sponge-associated 16S rRNA reads. These OTUs encompassed a cocktail of as-yet uncultivable phylotypes in the dominant phyla mentioned above, besides less-abundant lineages belonging to *Nitrospirae* and the candidate groups PAUC39f, SBR1093, and AncK6. All these OTUs could be considered typical *S. officinalis* endosymbionts not only because of their high abundance but also sharp enrichment in numbers within the host in comparison with the environment (**Figure 3** and Supplementary Tables S2, S3). Remarkably, this highly selected group of symbionts consistently displayed, with only a few exceptions, greater residual abundances in sediments than in seawater (Supplementary Table S3). Particularly, OTUs 40 and 37, representing uncultured lineages in the (*Acidimicrobiales Actinobacteria*) and Sva0725 (*Acidobacteria*) clades, ranked among the top-25 most abundant OTUs of the complex sediment communities (Supplementary Table S3). In addition, the three most dominant *S. officinalis* gammaproteobacterial symbionts (OTUs 621—order *Chromatiales*, 690—order *Thiotrichales*, and 639—order HTCC2188) displayed equivalent or even higher abundances in sediments (Supplementary Table S3). Conversely, the very dominant OTUs in seawater, essentially representing a mix of *Flavobacteriia*, *Alphaproteobacteria*, and *Gammaproteobacteria* phylotypes, were all markedly de-selected in the sponge host except for OTU 442 (uncultured *Rhodobacteraceae*, the second most abundant seawater phylotype), which was the 5th and 14th most abundant OTU in sediments and sponges, respectively (Supplementary Table S3).

### IPR Functional Profiling

**FIGURE 4:**
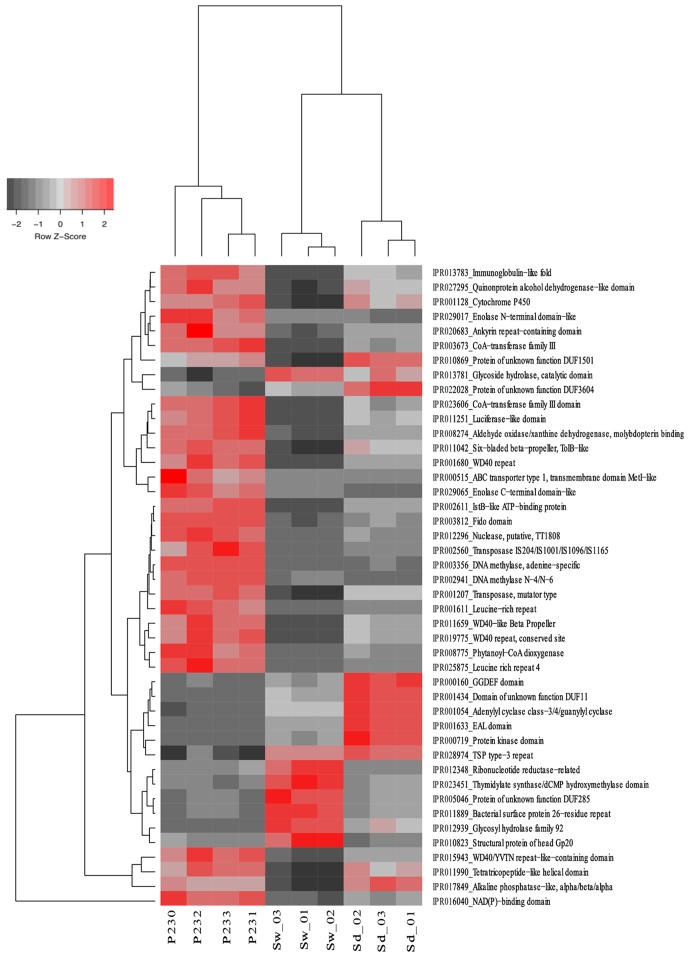
Heat map of the 44 most differentiating IPR entries across biotopes. The dendrogram clusters IPR entries according to their abundance distributions across biotopes, labeled at the bottom of the diagram. Heat map details are as in legend to **Figure 2**.

**FIGURE 5:**
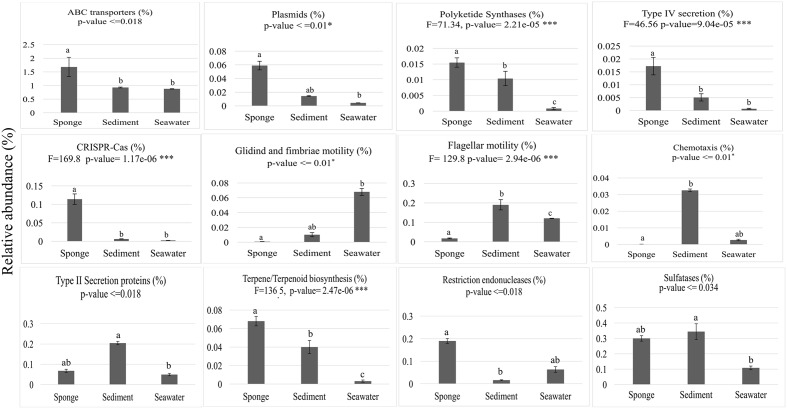
Abundance distributions of broad functional categories across biotopes. Values on the *y*-axis represent mean cumulative IPR relative abundances (%) in each biotope ± standard deviations. ABC transporters—19 IPR entries used in plot construction; Plasmids—10 IPR entries; Polyketide synthases—1 IPR entry; Type IV secretion—6 IPR entries; CRISPR-Cas—43 IPR entries; Motility—8 IPR entries involved in gliding and fimbriae-based motility; Flagellum, 56 IPR entries involved in flagellum assembly and motility; Chemotaxis—5 IPR entries; Type II secretion proteins—13 IPR entries; Terpene/Terpenoid biosynthesis—3 IPR entries; Restriction endonucleases—68 IPR entries; Sulfatases—4 IPR entries. All IPR entries can be identified in Supplementary Table S4. Results of the general test for differences among biotopes are shown at the top of each chart, below the label of each analyzed function. One-way ANOVA with *F* statistics results are shown for normally distributed data, whereas ANOVA on ranks results are shown for data distributions that did not pass normality tests. Bars labeled with different letters represent statistically distinct biotopes in terms of IPR relative abundances according to *post hoc* pairwise tests of significance.

From the 10,272 IPRs detected throughout the functional dataset using the EMG data processing pipeline (see section “Materials and Methods” for details), 6,046 were present in all biotopes, whereas 234, 695, and 1,130 were specific to *S. officinalis*, seawater, and sediments, respectively. However, 8,325 IPRs displayed significantly different (*p* < 0.05) abundance values (normalized data) among at least two biotopes (Supplementary Table S4), further substantiating the disparate functional assembly among the studied microbiomes (**Figure 1**). Due to the high complexity of the functional profiles and the thousands of IPR entries found to vary among biotopes, we used SIMPER analysis to rank those IPRs contributing the most to the total dataset variation (Supplementary Table S4). A heat map of the 44 IPR entries varying the most across the biotopes, found to explain >5% of (normalized) IPR abundance oscillations altogether, is shown (**Figure 4**). This group comprised several IPR entries contrasting the ecological and evolutionary contexts of the surveyed biotopes. Several functional traits strongly selected in the *S. officinalis* microbiome could be pinpointed, the majority of which showing higher residual abundances in sediments than in seawater. These included a series of ELP repeats (namely, WD40, leucine-rich, tetratricopeptide, and ankyrin repeats, in this order) which remarkably populated the top-oscillating IPRs list (**Figure 4** and Supplementary Table S4), along with luciferase-like, TolB-like beta propeller, ABC-transporter type 1, several transposases, and cytochrome P450 and CoA transferase III domain entries, among others (**Figure 4**). Worth mentioning among IPR entries more abundant in sediments were the GGDEF and EAL domains involved in synthesis and degradation of cyclic di-guanylate (c-di-GMP), known to regulate key cell physiology and life-style features such as motility, biofilm formation, and virulence factors. Manual inspection of thousands of IPR entries contributing significantly to data variation (Supplementary Table S4) allowed us to single out a number of “umbrella” functions (each encompassing several IPR entries) presenting sharply different abundances among the biotopes (**Figure 5** and Supplementary Figure S2). This approach clearly depicted the collective enrichment, in the *S. officinalis* microbiome, of all IPR entries classified into the above-mentioned ELPs (Supplementary Figure S2), and those representing the coding of CRISPR-Cas, restriction endonucleases, plasmids, polyketide synthases, terpene/terpenoid synthases, type IV secretion proteins and ABC transporters (**Figure 5**). Most of the observed sponge-enriched functional attributes showed, to varying degrees, significantly higher abundances in sediments than in seawater (**Figure 5** and Supplementary Figure S2), except for the ABC transporters category, which includes both import and export transporters, and the restriction endonucleases category, more abundant in seawater than in sediments. Particularly, we uncovered striking diversity of both CRISPR-Cas and restriction endonuclease cds from the *S. officinalis* microbiome (42 and 50 IPR entries, respectively). Restriction endonuclease reads represented, collectively, about 0.19% of the total number of annotated reads from *S. officinalis*, exceeding the relative abundance of CRISPR-Cas elements (0.11%) in these samples. Highly abundant in both sponge and sediment metagenomes were sulfatases, involved in the utilization of organic sulfated compounds, whereas type II secretion proteins involved in virulence were pronouncedly enriched in sediment metagenomes (**Figure 5**). Finally, predicted proteins involved in motility and chemotaxis were much more prevalent in sediments and seawater than in *S. officinalis* (**Figure 5**). While gliding and fimbriae types of motility were abundant in seawater, flagellar motility traits were more abundant in sediments.

### Functional Conservation among *S. officinalis* and Other Sponge Hosts

To verify the extent to which the microbial metagenome of *S. officinalis* resembles those of other sponge hosts regarding their functional attributes, we used MG-RAST to compare the COG profiles obtained in this study (using metagenome reads assembled with MetaVelvet—see Supplementary Appendix S1) with those retrieved by Fan et al. (2012) for *R. odorabile*, *C. concentrica*, and *C. coralliophila*. In spite of the large geographical distance between sampling sites and of the different sampling, sequencing, and data processing methods utilized in both studies, ordination analysis revealed a gradient in COG functional profiles corresponding to the phylogenetic relatedness of the hosts, with *S. officinalis* and *R. odorabile* (order Dictyoceratida) being placed closer to one another in the ordination diagram and farther apart from *C. concentrica* and *C. coralliophila* (order Axinellida) (Supplementary Appendix S1). The functional profiles of marine sponges, when pooled into one major group, differed significantly from those of seawater and sediment microbiomes. A high degree of functional conservation, at the COG-level, was observed among the sponge hosts, with 62.7% of all COGs listed being shared by the four species. Furthermore, sponges were collectively found to share more COGs in common with sediments than with seawater (Supplementary Appendix S1). SIMPER analysis (Supplementary Table S5) revealed that several of the common, enriched sponge symbiont functions were re-verified to contribute sharply to distinguish sponge from seawater and sediment metagenomes as observed in the analysis of *S. officinalis* IPR profiles. Particularly relevant in this regard were restriction-modification systems (i.e., restriction endonucleases), site-specific and adenine-specific DNA methylases, ABC transporters and plasmid-maintenance systems (Supplementary Table S5). As determined in the analysis of IPR profiles, sulfatases were abundant in both sponge and sediment metagenomes, whereas type II secretion proteins were more abundant in sediments (Supplementary Table S5).

### All Domains–All Genes Taxonomic Profiling Using MG-RAST

Within MG-RAST, we performed a taxonomic assessment, primarily at the domain level, taking all gene reads (and not only 16S rRNA gene reads) that could be taxonomically classified into account, enabling us to determine the distribution of major groups (i.e., domains and viruses) across the biotopes in a more comprehensive fashion. In all biotopes, bacteria were clearly the most dominant group, comprising over 95% of all classifiable gene reads (Supplementary Figure S3). While archaea were less represented in seawater (*c.* 0.18% of classifiable reads) than in sponges (1.6–3.9%) and sediments (1.7–3.2%), eukaryotic reads were slightly more abundant in the former biotope (3.11–4.36% of classifiable reads) than in the latter (2.12–2.39 and 2.04–2.32% in sponges and sediments, respectively). In spite of their minor representativeness across the entire dataset in terms of read numbers, from among all analyzed groups, viruses were found to oscillate the most in relative abundance among biotopes, displaying up to 13-fold higher abundances in seawater than in sponge and sediment samples (Supplementary Figure S3).

## Discussion

The taxonomic distinctiveness of the *S. officinalis* symbiotic consortium in comparison with those from its neighboring biotopes can be readily observed at the phylum level (**Figure 2** and Supplementary Table S2). Consistent with primer-based studies undertaken for other keratose sponges off the Algarve coast (Hardoim and Costa, 2014; Hardoim et al., 2014) and also from the Mediterranean Sea (Erwin et al., 2012a; Pita et al., 2013) and the Great Barrier Reef (Webster et al., 2010), this community is primarily made of a complex mix of so-far uncultivable, sponge-enriched symbiotic bacteria. Owing to our comparative experimental design, we gathered compelling evidence for higher sponge symbiont abundances in sediments than in seawater, revealing an unexpected pattern of distribution of these microorganisms across marine biotopes and extending previous knowledge gained on their occurrence, at very low abundances, in the bacterioplankton (Webster et al., 2010; Webster and Taylor, 2012). Particularly, we identified one possible “generalist par excellence” bacterium in the *Rhodobacteraceae* clade (OTU 442) which, although clearly being a profuse member of seawater communities, likely performs well both in sediments and sponges. Further, the high prevalence of sponge-enriched *Acidobacteria* (especially Sva0725 phylotypes), *Actinobacteria* (*Acidimicrobiales* phylotypes), and *Gammaproteobacteria* (several different orders) in sediments adds further layers of complexity to our understanding of sponge symbiont occurrence in the marine realm. Future cultivation-independent, genome-wide studies targeting the adaptive features of these lineages not only hold promise in revealing their likely roles in the sponge endosymbiotic consortium, but may also improve our view of the genetic traits underpinning the persistence of sponge symbionts in the open environment, and consequently of the evolutionary and ecological forces that mediate the dispersal and community assembly of marine sponge symbionts. However, specific studies aiming at uncovering the potential metabolism, linking identity and function, of foundational sponge-associated bacteria are still relatively scarce. SCG and cultivation-independent genome binning from metagenomes have been proven useful in this regard, unveiling, e.g., halogenation capacities within sponge-associated *Chloroflexi*, *Actinobacteria*, and *Poribacteria* spp. (Bayer et al., 2013), non-ribosomal peptide biosynthesis potential within the *Chloroflexi* (Siegl and Hentschel, 2010) and multiple adaptive features of the keystone sponge-associate cyanobacterium *Synechococcus spongiarum* (Gao et al., 2014; Burgsdorf et al., 2015). Recently, the ability of several, so-far uncultivable sponge symbionts to utilize carnitine, a quaternary ammonium compound regularly present in the mesohyl matrix of sponges, has been revealed, suggesting parallel adaptation of multiple lineages to a common resource within the *in-spongia* microniche (Slaby et al., 2017). Our taxon-independent, primer-less sequencing approach revealed a pronounced dominance of one *Poribacteria* OTU in *S. officinalis*. It is therefore reasonable to argue that some of the potential metabolic features recently revealed for poribacterial symbionts by means of SCG (Siegl and Hentschel, 2010; Siegl et al., 2011; Kamke et al., 2013) are likely to mediate major bioprocesses and molecular interactions within the *S. officinalis* endosymbiotic consortium. These features include, among others, polyketide biosynthesis capacities (possibly involved in host’s chemical defense), a vast, specialized carbohydrate degradation repertoire (considered pivotal to host’s nutrient provision), and enrichment of eukaryotic-like repeat proteins (e.g., TRPs, ANKs, LRRs, usually considered to enable symbionts to evade phagocytosis by the host) all of which could be verified, from our community functional profiles, as characteristic of the *S. officinalis* microbial metagenome. Because these attributes have been commonly verified in diverse sponge symbiont lineages (Slaby et al., 2017), it can be argued that they contribute significantly to the observed difference in taxonomic assembly between sponges, sediments, and seawater observed here.

One important finding in this study was the observation that several of the features identified as genomic signatures of the *S. officinalis* microbiome displayed higher abundances in sediments than in seawater. Among these traits we highlight IPR entries underlying the coding of an array of ELPs or involved in plasmid assembly, stability and conjugative transfer (e.g., plasmid replication, toxin–antitoxin systems and type IV secretion IPRs), secondary/cytotoxic metabolite biosynthesis (e.g., polyketide and terpene/terpenoid synthases, TolB-like and cytochrome P450 IPRs), remediation of oxidative stress (a luciferase-like domain), organic carbon utilization (e.g., sulfatases) and literally hundreds of other individual IPR entries (**Figure 5**, Supplementary Figure S2, and Supplementary Table S4). Therefore, some of the features previously regarded as “unique” adaptations of the sponge symbiotic consortium may be well represented in other marine settings. Below, we give emphasis to the above-mentioned functions and discuss their patterns of occurrence across bacterial genomes and the marine biotopes studied here.

Inspection of the IPR entries contributing the most to variation in the functional dataset (**Figure 5**) revealed the consistent prevalence of ELPs (TRPs, ANKs, LRRs, and WD40) among the most sensitive IPRs differentiating the studied biotopes, all of which were enriched in *S. officinalis* (Supplementary Figure S2). The abundance of TRPs and ANKs in sponge microbiomes has been well documented (Thomas et al., 2010; Fan et al., 2012), and a role for these ELPs in preventing phagocytosis of bacterial symbionts by the sponge host has been proposed (Nguyen et al., 2014; Reynolds and Thomas, 2016). In the present study, contrary to previous reports addressing other sponge hosts (Thomas et al., 2010; Fan et al., 2012), WD40 repeats were by far the most abundant ELP repeat in the *S. officinalis* microbiome, with several entries varying markedly in abundance across the surveyed biotopes (**Figure 4** and Supplementary Table S4). WD40 repeats are regarded as prevalent in eukaryotes and uncommon in prokaryotes, and act as a protein–protein or protein–DNA platforms to allow for various protein complex assemblies in cellular metabolism (Xu and Min, 2011; Wang et al., 2015). However, evidence from this study and elsewhere (Díez-Vives et al., 2016; Reynolds and Thomas, 2016) is now accumulating for a broad distribution of these motifs among sponge symbiotic bacteria, offering a new angle from which the spread of these macromolecule network hubs can be seen throughout the tree of life. Collectively, the presence of ELPs in prokaryotic genomes has been interpreted as suggestive of lateral host–microbe gene transfer given their presumed eukaryotic origin (Horn et al., 2016). Recently, ELPs were shown to be positively expressed in sponge microbial metatranscriptomes (Díez-Vives et al., 2016), supporting their likely importance in mediating cell–cell interactions within the sponge holobiont. In particular, the expression of WD40 repeats was found to be associated with domains of the Tol-dependent translocation system, which is involved in outer membrane integrity, cell invasion and, eventually, pathogenesis of Gram-negative bacteria, suggesting a pivotal role of these repeats in host–microbe interactions (Díez-Vives et al., 2016). The enriched abundance of both WD-40 repeats and one TolB-like domain (IPR011042) in the *S. officinalis* microbial metagenome speaks for distinguishing host colonization capacities and/or virulence potential within this symbiotic consortium.

Polyketides have been intensively studied as sponge-derived natural products whose biosynthesis is primarily mediated by bacteria (Piel, 2002; Piel et al., 2004; Wilson et al., 2014), and are thought to play a role in defense of the sponge host against natural enemies, as demonstrated for the bryozoan host *Bugula neritina* (Lopanik et al., 2004). Terpenes and terpenoids, in their turn, encompass a large class of natural products commonly regarded as of fungal and plant origin whose biosynthesis by bacteria is attracting increasing research interest (Yamada et al., 2015). The most abundant IPR entry related with terpene/terpenoid biosynthesis in *S. officinalis* (IPR008930) corresponds to a family of terpenoid cyclases/protein prenyltransferases responsible for a wide chemodiversity of terpenoid natural products (Christianson, 2017). Considering the broad distribution of terpene/terpenoid systhase genes across bacterial genomes (Yamada et al., 2015), it is tempting to argue that terpenoid biosynthesis in *S. officinalis*, and marine sponges in general, could be as well mediated by bacterial symbionts, emerging as a further mechanism possibly conferring host defense against natural enemies or mediating microbe–microbe interactions within the sponge host. Likewise, cytochrome P450 enzymes (IPR001128) are a superfamily of monooxygenases presenting broad substrate spectrum, being widespread in all domains of life. Particularly in bacteria, they are important in the biosynthesis of secondary metabolites such as erythromycin, and bear potential for applications in synthetic biology and the pharmaceutical industry (Girvan and Munro, 2016). Taken together, these observations suggest high microbially driven chemical complexity within the *S. officinalis* holobiont. Such a vast secondary metabolite repertoire may play pivotal roles in microbiome community assembly, host–symbiont signaling, and host defense. Widely known for their key role in bioluminescence, bacterial luciferases are flavin monooxygenases which incorporate or reduce molecular oxygen in redox reactions, and may have originally evolved as enzymes responsible for reactive oxygen species detoxification (Szpilewska et al., 2003). The sharp enrichment of this trait in the *S. officinalis* microbiome, followed by sediments, leads us to posit that these enzymes may primarily act as anti-oxidant agents in these particular settings, along with other anti-oxidant enzymes known to be enriched in sponges such as glutathione peroxidases (Thomas et al., 2010), observed here to possess high abundance in both sponges and sediments (Supplementary Table S4, IPR IPR000889). Enrichment in sulfatases/aryl sulfatases have been suggested as a specialization of marine sponge symbionts enabling them to utilize sulfated polysaccharides from the host’s extracellular matrix (Slaby et al., 2017). Sulfatase-encoding genes were abundant not only in the *S. officinalis* (Supplementary Table S4 and **Figure 5**) metagenome, but ranked as one major genetic signature of several sponge-associated microbiomes (Supplementary Table S5). Here, we reveal that this trait is equivalently enriched in both sponge and sediment biotopes in comparison with seawater, providing evidence for the common selection of fundamental nutrient acquisition capacities in phylogenetically contrasting microbiomes. In the context of the marine sponge holobiont, sulfatases are supposed to be involved in nutritional exchange between host and microbes, playing a vital role in the cycling of sulfur within the animal.

Altogether, the outcomes delineated above indicate closer resemblance in functional attributes between sponges and sediments than sponges and seawater: a hypothesis corroborated by Bray–Curtis dissimilarity measures calculated for the three biotopes based on the whole array of 10,272 IPR entries uncovered from the data (**Table 1**). However, the quantitative trend revealed with whole functional profiles must be considered with caution since statistical significance varied depending on data processing methodology and on the reference database employed (Supplementary Appendix S1). Importantly, the level of phylogenetic disparity between all microbiomes was high (**Table 1**) in spite of our observation for higher residual symbiont abundances in sediments than in seawater (see above). Therefore, it is likely that surface sediments and endosymbiotic sponge communities, although being chiefly composed by different microbial populations (especially regarding their very dominant members) display a certain degree of independent functional convergence. This prompts us to argue that selective pressures common to particle- and host-associated modes of living constitute an important evolutionary force shaping functional assembly in marine biomes. Several factors, ranging from cell–cell interactions to availability (in quality and quantity) of solid surfaces for cell attachment to modes of symbiont acquisition and release by sponges, may contribute to the observed trends. Microbial cell densities alone, known to be about three orders of magnitude higher in coastal sediments (Schmidt et al., 1998) and in Dictyoceratida sponges (Hardoim et al., 2012) than in seawater, may be a key factor promoting genetic exchange and adaptive features likely to prevail in the former biotopes. In highly dense circumstances, gene clusters involved in the biosynthesis of natural products such as polyketides and terpenes—or more specifically terpene-quinones very often enriched in Dictyoceratida species such as *S. officinalis* (Gordaliza, 2010; Manzo et al., 2011; Li et al., 2017)—are likely to confer selective advantage to its carriers. Similarly, strategies to neutralize cytotoxic effects are likely to elicit a selective advantage in communities where inhibitory compounds abound. ABC transporters (**Figure 5**) comprise a large family of bacterial trans-membrane proteins mediating the import and export of small and large-sized molecules throughout the cell, and may play a fundamental role as detoxifying agents permitting microbial survival in competitive microniches. Particularly, we found permeases within the ABC transporter category (e.g., IPRs 001851, 0038381, and 025966, Supplementary Table S4) with presumed, manifold detoxifying functions commonly abundant in sponges and sediments. Polyketide synthases, type IV secretion and ABC transporter-encoding genes have all been detected on plasmids from several microorganisms (Stinear et al., 2004; Kadlec and Schwarz, 2009; Bruto et al., 2017). The prevalence of these genes along with the higher incidence of plasmid, transposase and ELP-encoding genes (which by themselves speak for greater genetic exchange potential) in the *S. officinalis* microbiome, followed by sediments, hints at a possible convergent selection of these traits in phylogenetically divergent microbial communities. Future studies aiming to define the gene content of the community of circular plasmids present in marine sponges will certainly shed new light on the functional features more likely to traffic about in the mobile gene pool within the Porifera.

Physical connectivity between sponges and sediments, although usually given less importance in microbiology studies than seawater intake via filtering, takes place by the capture of particulate organic matter and particles in suspension by the sponge host (Schönberg, 2016). In addition, loss of sponge cells through shedding and sponge-expelled detritus, both found to be significant processes in sponge cell turnover (Alexander et al., 2014), may act as substantial inputs of sponge-associated microorganisms into superficial sediment layers. Thus, marine sediments may serve as both sources and sinks of sponge-associated microorganisms, but the magnitude and relevance of this exchange remains to be addressed. The moderate abundance of a few dominant sponge symbionts in sediments indicates that these bacterial lineages, if not optimal performers, are capable of persisting—for undetermined periods—at considerable densities in this alternative habitat, thereby enhancing their probability of future lateral acquisition by the sponge host. Identifying an active role beyond environmental endurance for these lineages in the complex microbiome of marine sediments is challenging. It is known that several factors such as seawater temperature and the composition and age of biofilm and biofouling communities are decisive for invertebrate larval settlement in benthic ecosystems (Hadfield, 2011; Whalan and Webster, 2014). Therefore, it could be argued that increased inter-domain signaling between host larvae and a seeding community of competent sponge associates on particulate/hard substrate—or microbe–microbe signaling in such circumstances—may contribute to higher larval settlement rates in favorable microniches, promoting the selection, on the sea floor, of sponge symbiont lineages able to persist in the open environment.

Contrasting the trends discussed above, microbial genes involved in motility and chemotaxis were altogether more prevalent in seawater and sediment communities, and much less abundant in the sponge host. The ability to move and orchestrate movement in response to chemical cues and gradients are widely acknowledged as imperative mechanisms dictating the distribution of microorganisms in the oceans (Stocker and Seymour, 2012) and as quintessential features of host-associated bacteria (Wadhams and Armitage, 2004; Rawls et al., 2007). Here we show that the *S. officinalis* endosymbiotic consortium displays low abundance of genomic features involved in chemotaxis and flagellar, gliding and fimbriae-based motilities when compared to its surrounding environment, supporting the idea that loss of motility may be common among prevalently vertically transmitted symbionts (Bright and Bulgheresi, 2010). Or, alternatively, for symbionts whose mode of acquisition by the host is rather passive from the microbial standpoint. Particularly relevant in distinguishing sediments from sponges and seawater regarding the regulation of virulence and motility were the higher abundances of GGDEF and EAL protein domains and of type II secretion proteins in sediments. The above-mentioned domains modulate the concentrations of cellular c-di-GMP, a signaling molecule involved in the regulation of biofilm formation, virulence, motility and cell surface adhesiveness in Gram-negative bacteria (Argenio and Miller, 2004). Indeed, increased cellular c-di-GMP was found to promote type II secretion activity in *Vibrio cholerae* (Beyhan et al., 2006). Therefore, signal transduction via c-di-GMP and its modulation appears to be a determining factor in shaping the virulome of marine sediments in a singular fashion. Considering the *S. officinalis* endosymbiotic consortium, it is likely that this community essentially consists of “sit-and-wait” performers regarding their nutrient foraging strategies, especially if it is assumed that filtering activity alone is responsible for the total import and distribution of organic carbon and energy into the host. Our dedicated sampling of the inner sponge body disregards the profuse and complex community of epibionts known to populate the pinacoderm of keratose sponges, where photosynthetic Cyanobacteria are favorably selected (Erwin et al., 2012b) and motility and chemotaxis traits may be relevant for colonization and biofouling processes. The consistent trend found here for a primarily heterotrophic, less-motile community of endosymbionts highlights the need of approaching distinct microniches within marine sponges for a better understanding of microbiome spatial distributions and dynamics in these hosts (Webster and Thomas, 2016).

Finally, we detected much higher incidence of CRISPR-Cas and restriction endonucleases in the *S. officinalis* microbiome than in seawater—in accordance with earlier metagenomics surveys (Thomas et al., 2010; Fan et al., 2012; Horn et al., 2016)—and sediments. Therefore, in the context of its two immediate environmental surroundings, the enrichment of both defense mechanisms can be indeed considered a true hallmark of the *S. officinalis* microbiome, and much likely of marine sponges in general. Much has been discussed on the diversity (Fan et al., 2012; Horn et al., 2016) and role of these genetic elements as an efficient, specific anti-phage defense system permitting bacterial survival within the sponge microbial consortium (Thomas et al., 2010; Fan et al., 2012; Horn et al., 2016). In agreement with this hypothesis, we here observed that the relative abundance of both defense systems and of bacteriophages were inversely correlated in *S. officinalis* and seawater, where viral particles were 13-fold more frequent than in sponges and CRISPR-Cas were virtually absent. However, low abundances of both defense systems and of viral DNA were detected in sediments, suggesting that viral populations might be regulated by other mechanisms in these settings rather than high abundance of CRISPR-Cas and R-M systems alone. In this regard, it was curious to note that the diversity and assemblage of restriction endonucleases uncovered from sponges and sediments was fairly comparable, with higher abundances in sponges being the primary factor distinguishing these biotopes (Supplementary Table S4). Future efforts are therefore needed to disentangle the relative forces exerted by CRISPR-Cas and restriction-modification systems on the regulation of viral populations within the Porifera and across marine biomes. To this end, better understanding of the structure of phage communities in host- and particle-associated settings will be much required.

## Conclusion

In conclusion, the comprehensive comparative metagenomics strategy employed in this study enabled us to critically assess the distribution of genomic features involved in symbiosis across marine habitats, and to address functional convergence vs. divergence in contrasting marine microbial communities more thoroughly. We advocate that such an approach, which in the future shall include the assessment of other invertebrate hosts, is imperative for a holistic understanding of microbial community dynamics and function in marine sponges and benthic ecosystems at large.

## Ethics Statement

This study relied on *in situ* sampling of microorganisms from marine invertebrates without a nervous system, and as such was exempt from ethical approval procedures according to the current Portuguese legislation (Decreto-Lei n° 113/2013). This study did not occur within privately owned or protected areas. This study did not involve endangered or protected species. The sampling methodology privileged minimally invasive handling procedures, following the guidelines of the European Directive 2010/63/EU.

## Author Contributions

RC designed the study. EK, JG, JX, and RC performed the experiments. MPR, JG, JX, and RC provided reagents and materials. EK, MR, and RC analyzed the data. EK and RC wrote the main manuscript text and prepared figures and tables. All authors reviewed the manuscript.

## Conflict of Interest Statement

The authors declare that the research was conducted in the absence of any commercial or financial relationships that could be construed as a potential conflict of interest.

## Footnotes

1 https://www.ebi.ac.uk/interpro/release_notes_31.0.html

2 http://bioinfogp.cnb.csic.es/tools/venny/

3 https://www.ncbi.nlm.nih.gov/

4 http://www.ebi.ac.uk/ena
