# Supplementary material for: Comparative Metagenomics Reveals the Distinctive Adaptive Features of the Spongia officinalis Endosymbiotic Consortium
Source: Front Microbiol. 2017 Dec 14;8:2499. doi: 10.3389/fmicb.2017.02499 (PMC5735121; doi:10.3389/fmicb.2017.02499)
Supplement: Supplementary file 6 [file Data_Sheet_2.DOCX]

Supplementary Material

**Comparative metagenomics reveals the distinctive adaptive features of the *Spongia officinalis* endosymbiotic consortium**

Elham Karimi^1^, Miguel Ramos^1$^, Jorge M.S. Gonçalves^2^, Joana R. Xavier^3^, Margarida P. Reis^4^, Rodrigo Costa^1,5*^

*** Correspondence:** Rodrigo Costa: [rodrigoscosta@tecnico.ulisboa.pt](mailto:rodrigoscosta@tecnico.ulisboa.pt)

# Supplementary Data

**Appendix S1 │ Preliminary data analyses and data validation with alternative analytical pipelines**

# Supplementary Figures and Tables

## Supplementary Figures

**Figure S1 │Cytochrome oxidase I (COI) gene-based phylogenetic inference of sponge specimens examined in this study and their closest relatives.** All sequences were aligned within the software package MEGA7. The evolutionary history was inferred using the Maximum Likelihood method based on the Kimura 2-parameter model. The tree for the heuristic search was obtained by applying the Neighbor-Joining method to a matrix of pairwise distances estimated using the Maximum Composite Likelihood (MCL) approach. A discrete Gamma distribution was used to model evolutionary rate differences among sites. Percent bootstrap values greater than 70% are shown. The tree is rooted to the *Rhopaloeides* genus and sponges from the present study are shown in bold.

**Figure S2│Abundance distributions of ELPs (TRPs, ANKs, LRRs and WD40) across biotopes.** Values on the y-axis represent mean cumulative IPR relative abundances (%) in each biotope ± standard deviations. Ankyrin repeats - 3 IPR entries used in plot construction; Tetratricopeptide repeats - 10 IPR entries; WD40 repeats - 4 entries; leucine-rich repeats - 5 IPR entries. Results of the general test for differences among biotopes (One-Way ANOVA) are shown at the top of each chart, below the label of each analyzed function. Bars labeled with different letters represent statistically distinct biotopes in terms of IPR relative abundances according to pair-wise tests of significance.

**Figure S3│Relative abundance and distribution of microbial domains (A) and viruses (B) across the biotopes based on best-hit classifications using MG-RAST.**

## Supplementary Tables

**Table S1 │Number of sequence reads per quality control steps using the EBI metagenomics (EMG) pipeline (v. 2.0).**

**Table S2 │16S rRNA gene-based distribution of microbial phyla (A) and OTUs (B) across biotopes.**

**Table S3 │Most differentiating microbial phyla (A) and OTUs (B) among biotopes.**

**Table S4** │**Most differentiating IPR entries among biotopes.**

**Table S5 │ Most differentiating COG entries among biotopes, with "sponges" representing functional profiles of *S. officinalis*, *R. odorabile*, *C. concentrica* and *C. coralliophila*.**
