# Supplementary material for: Comparative Metagenomics Reveals the Distinctive Adaptive Features of the Spongia officinalis Endosymbiotic Consortium
Source: Front Microbiol. 2017 Dec 14;8:2499. doi: 10.3389/fmicb.2017.02499 (PMC5735121; doi:10.3389/fmicb.2017.02499)
Supplement: Supplementary file 7 [file Data_Sheet_3.DOCX]

**Appendix S1 │ Preliminary data analyses and data validation with alternative analytical pipelines**

**Metagenome data assembly**

Metagenome assembly was performed using MetaVelvet (Namiki et al., 2012) under default settings. This procedure enabled us to obtain higher numbers of assembled nucleotides from complex sediment metagenomes than assembly using IDBA_UD, whereas performance of both assemblers was comparable for seawater and sponge metagenomes (data not shown). Still, because of the low proportions of reads actually used in the generation of contigs, and the low average contig length obtained for sediment samples (**Table AS1.1**), we opted to perform our core comparative metagenome analysis using unassembled data (see Figs. 1 to 5 in the main text, and Figs. S2 and S3 in the supplements). Metagenome assemblies obtained with MetaVelvet were subjected to COG-based annotations. While COG profiles from both unassembled and assembled data were used to (1) verify whether patterns described for IPR profiles using unassembled data could be reproduced using other analytical pipelines, COG profiles from assembled data only were used to (2) compare the functional profiles obtained for the metagenomes analysed in this study with those published by Fan et al. (2012) to screen for common functions (i.e. COG entries) across different sponge hosts (see below).

**Table AS1.1 │ MetaVelvet assembly of *Spongia officinalis* (SP), sediment (Sd) and seawater microbial metagenome samples**

| **Sample** | **# contigs** | **Reads used (%)** | **N50 (bp)** | **Largest contig (bp)** | **Mean contig (bp)** | **Total assembly (bp)** |
| --- | --- | --- | --- | --- | --- | --- |
| SP230 | 122 435 | 51.51 | 631 | 45 968 | 510 | 62 460 207 |
| SP231 | 31 651 | 52.63 | 1 615 | 42 085 | 1 128 | 35 694 965 |
| SP232 | 152 240 | 51.75 | 385 | 70 453 | 393 | 59 774 639 |
| SP233 | 91 672 | 53.69 | 842 | 84 626 | 570 | 52 254 976 |
| Sd_01 | 1 437 527 | 19.10 | 97 | 1 203 | 76 | 109 116 486 |
| Sd_02 | 2 097 956 | 26.43 | 83 | 1 006 | 65 | 135 483 210 |
| Sd_03 | 2 858 973 | 28.35 | 88 | 1 151 | 60 | 171 780 628 |
| Seawater_01 | 759 222 | 68.95 | 320 | 22 151 | 102 | 77 133 569 |
| Seawater_02 | 75 462 | 35.63 | 600 | 18 025 | 320 | 24 150 712 |
| Seawater_03 | 68 013 | 32.23 | 666 | 35 041 | 344 | 23 372 397 |

**COG annotations of unassembled and assembled reads**

Within MG-RAST, we performed COG-based annotations for both the (1) unassembled dataset (also used in IPR annotations with the EBI metagenomics pipeline (EMG) - see main text) and (2) the assembled dataset obtained with MetaVelvet (**Table AS1.1**). In both cases, Principal Coordinates Analysis (PCoA) performed on the functional profiles recovered the overall trend observed with the analysis of InterPro (IPR) functional categories (see main text, Fig. 1), depicting highly contrasting, significantly different microbiomes in terms of function (p < 0.0006) across the three biotopes (**Fig. AS1.1**). Particularly, the sponge symbiotic consortium was found to significantly differ from seawater and sediment microbiomes for both assembled and unassembled datasets, whereas no significant, pairwise differences between seawater and sediment microbiomes were found (**Fig. AS1.1**). In contrast with results obtained using the IPR database (main text), COG annotations did not reveal the specific pattern of closer functional resemblance between sponges and sediments than between sponges and seawater (p > 0.05 for differences between Bray-Curtis dissimilarities). Thus, this particular outcome may slightly shift depending on the data processing pipelines and databases being used. Differently from the IPR annotation using EMG, COG annotations for both assembled and unassembled reads resulted in skewed numbers of reads with assigned functions among the different samples. Although the data transformation procedure used in this study corrects quite well for highly skewed data, it is important to consider this aspect when interpreting the results retrieved with COG annotations. Further, for all samples analysed, much higher numbers of reads could be assigned functions using the IPR database in comparison with the COG database (22,156,186 vs 4,559,625 reads with function across the whole unassembled dataset, respectively). Likewise, the total number of IPR entries uncovered from the whole unassembled dataset was as well much higher than the total number of COGs (10,272 IPR vs. 2497 COG entries). Altogether, these outcomes suggest that the use of the EMG processing pipeline resulted in a more refined annotation of our data due to both (1) higher equitability among the total number of reads analysed in each sample (see Table S1) and (2) much higher numbers of annotated reads computed along with higher diversity of functions (i.e. IPR entries) retrieved for all samples, substantiating our choice to use this particular analysis in our main results. Yet the COG annotations were very useful to contrast our data with COG-based profiles obtained previously for other sponge hosts, such as *Rhopaloides odorabile*, *Cymbastela concentrica* and *Cymbastela coralliophila*, all characterized by Fan et al. (2012).

**Contrasting functional profiles of different sponge hosts**

As mentioned above, functional COG profiles retrieved for metagenomes assembled in this study were compared with those retrieved by Fan et al. for the sponge hosts *Rhopaloeides odorabile* (belonging to the order Dictyoceratida, as *Spongia officinalis*), *Cymbastela concentrica* and *Cymbastela coralliophila* (belonging to the order Axinellida). In spite of the differences in sampling and sample processing procedures, next-generation sequencing methodology and throughput, data processing, and size of the metagenome libraries produced in this and in the Fan et al. (2012) studies, PCoA on Hellinger transformed data (for both non-rarefied and rarefied datasets) revealed a interesting gradient in functional profiles resembling the taxonomic relatedness of the sponge hosts, whereby *S. officinalis* and *R. odorabile* shared greater similarities with one another than with either of the *Cymbastela* hosts (**Fig. AS1.2**). When lumped together as one single group, marine sponge microbial metagenomes were found to possess functional profiles significantly different from those retrieved for the sediment and seawater metagenomes sequenced in this study (**Fig. AS1.2**). Here, the same trend observed for IPR profiles retrieved from unassembled reads could be gathered: marine sponges had, collectively, significantly greater similarity with sediments (average Bray-Curtis dissimilarity: 23.4%) than with seawater metagenomes (average Bray-Curtis dissimilarity: 26.1%) (p = 0.0042). However, it is worth mentioning, as explained above, that the different data generation methods and analytical pipelines may influence these results to some extent.

**COGs specific to and shared by *S. officinalis* and other sponge hosts**

Independently of the quantitative assessments highlighted above, our comparative scheme enabled us fetch those COG entries shared by and specific to each of the sponge metagenome libraries analysed. In line with PCoA results (**Fig. AS1.2**), we found that *S. officinalis* had more COGs in common with *R. odorabile* than with *C. concentrica* and *coralliophila* (**Fig. AS1.3**). Likewise, sponges altogether possessed more COGs in common with sediments than with seawater (data not shown). The functional core of the four sponge species was high (1691 COGs), representing 61.2% of all COGs identified in these metagenomes and revealing a considerable extent of functional convergence not only across a wide host phylogeny spectrum (as observed by Fan et al., 2012), but also geographical distances. Interestingly, SIMPER analysis of COG profiles listed for all four sponges together against sediment and seawater metagenomes revealed several sponge-enriched functions ranking as the most differentiating among biotopes (**Table S5**). Remarkably frequent among such top COG entries were type I and II restriction-modification systems identified here and by Fan et al. (2012) as sponge microbiome genetic signatures. Also, several of the observations made for IPR functional profiles obtained from unassembled reads could be revisited in this analysis, such as the higher abundance of ankyrin, tetratricopeptide, leucine-rich and WD-40 repeats in the sponge metagenomes, followed by sediments, as well as the distribution of polyketide, plasmid stabilization systems, ABC transporters and cytochrome P450 predicted functions, for instance, which followed the same trends observed for IPR annotations (**Table S5**).

**Figure AS1.1 │** **Principal Coordinate Analysis (PCoA) of functional microbial community profiles across biotopes based on COG annotations of unassembled (A) and assembled (B) metagenomes.** Community ordinations were based on pairwise Bray-Curtis dissimilarities calculated from normalized data, considering oscillations of relative COG abundances among samples. Analyses were performed on COG community profiles extracted from the metagenomes using MG-RAST. The first and second coordinates explain 39.7% and 36.1.% (A) and 51.2% and 24.6% (B) of the total dataset variation within unassembled and assembled metagenomes, respectively. Significance values resulting from permutational analysis of variance (PERMANOVA) applied to the corresponding dissimilarity matrices are as follows. Overall differences among groups: p = 0.0004 and 0.0005 for unassembled and assembled metagenomes, respectively. Pairwise significances: sponges were found to be different from seawater and sediment metagenomes in both datasets, with p values < 0.03 and < 0.04 for unassembled and assembled data, respectively. No significant difference was found between sediment and seawater functional profiles in both datasets (p > 0.05).

**Figure AS1.2 │** **Principal Coordinate Analysis (PCoA) of COG functional profiles obtained for microbial metagenomes assembled in this study and those obtained for other sponge hosts.** Community ordinations were based on pairwise Bray-Curtis dissimilarities calculated from normalized (Hellinger-transformed, non-rarefied) data, considering oscillations of relative COG abundances among samples. Ordination using data normalization after rarefying the metagenome libraries (standardization of all samples to the least sequenced sample) revealed the same trends as ordination using normalization on non-rarefied libraries libraries. Analyses were performed on COG community profiles extracted from the corresponding metagenomes using MG-RAST. *Rhopaloeides odorabile* (Rho), *Cymbastela concentrica* (Cyn) and *Cymbastela coralliophila* (Cyr) microbial metagenomes (Fan et al., 2012) were used in a comparative analysis against the COG-annotations retrieved in this study from *S. officinalis*, sediment and seawater metagenome assemblies. The first and second coordinates explain 34.3% and 17.7% of the total dataset variability, respectively. Significance values resulting from permutational analysis of variance (PERMANOVA) applied to the corresponding dissimilarity matrix are as follows. Overall differences among groups (all sponges vs. seawater vs. sediments): p = 0.0001. Pairwise significances: sponges were found to be different from seawater (p = 0.0057) and sediment (p = 0.0153) metagenomes, while no significant difference was found between sediment and seawater functional profiles (p = 0.309).


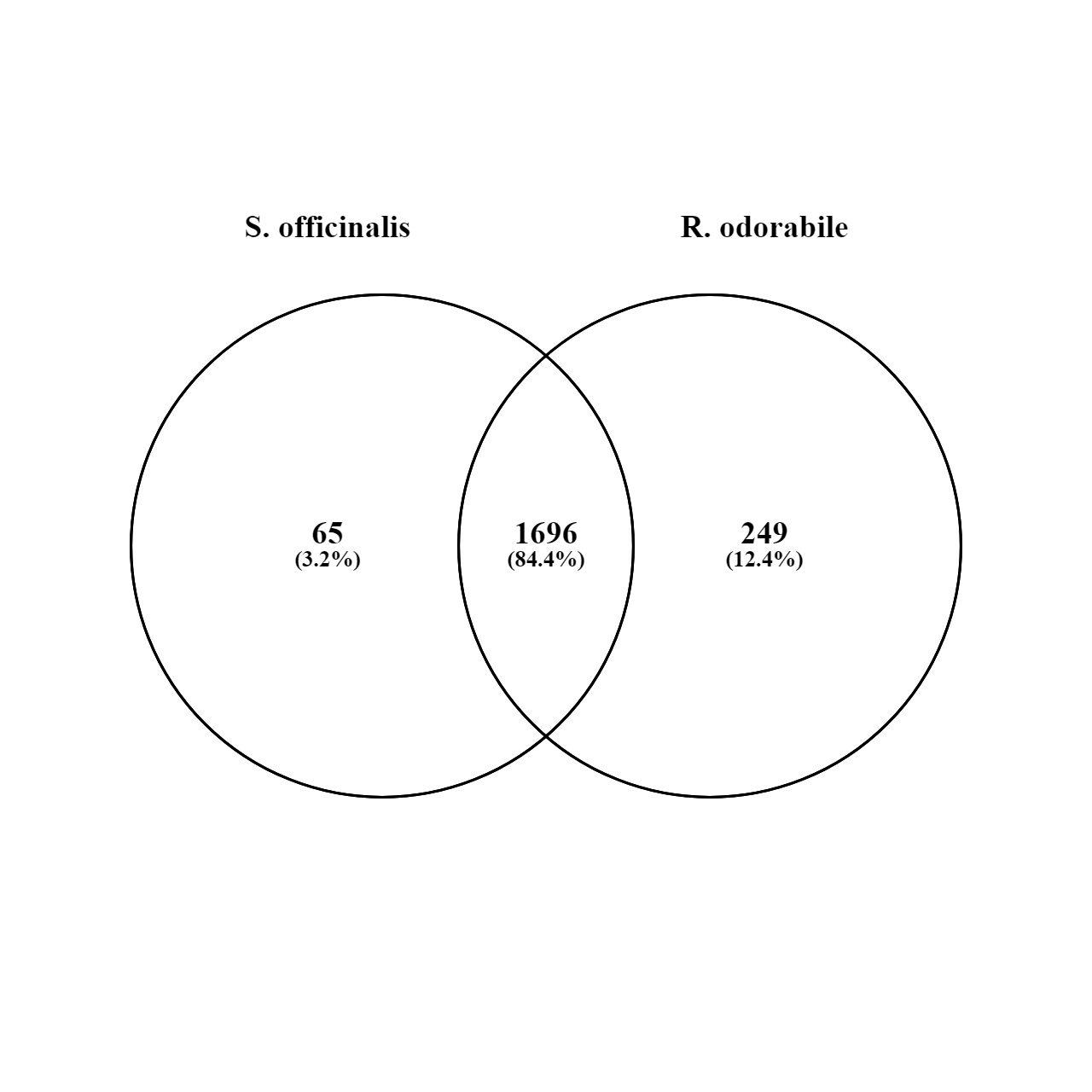

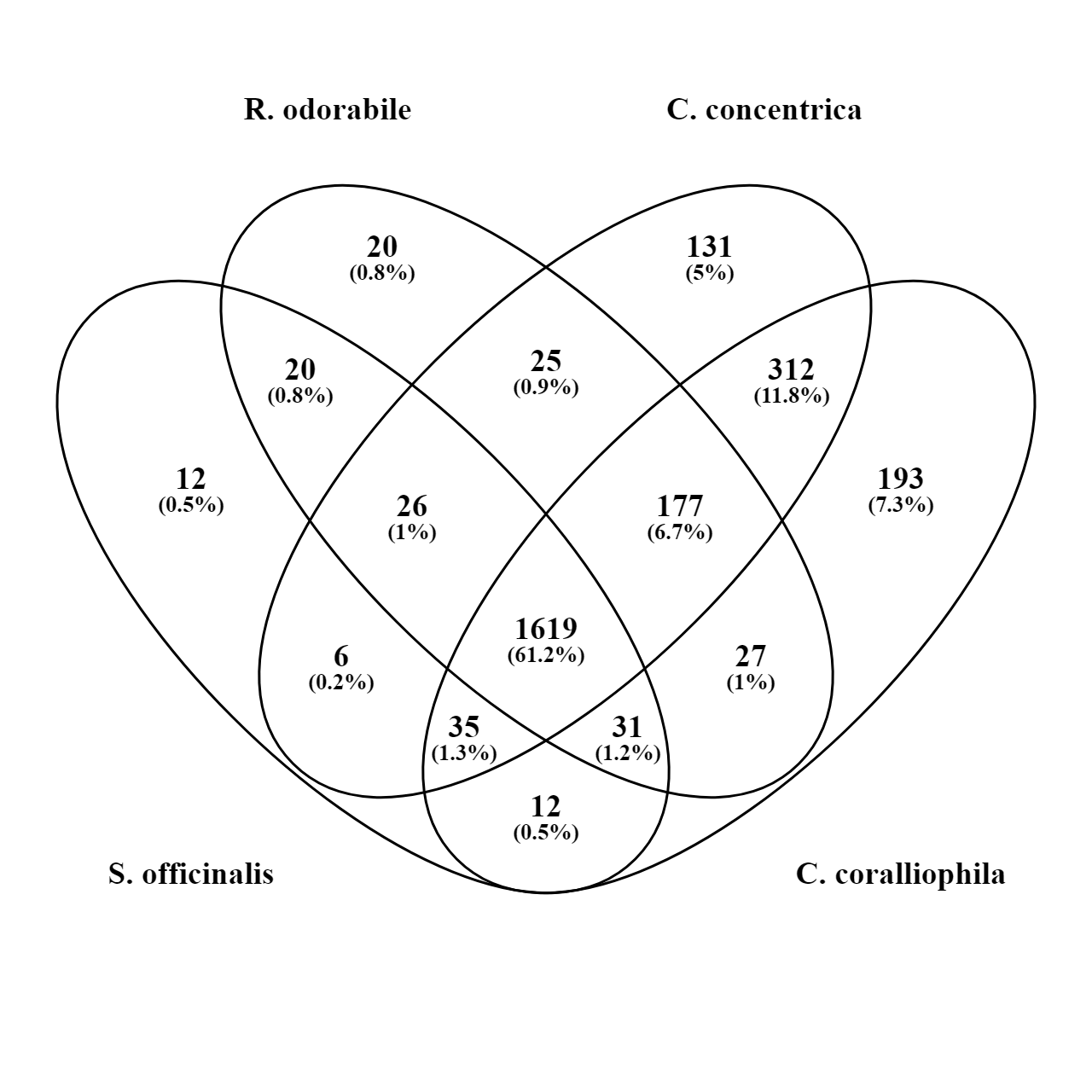


(B)

(A)

**Figure AS1.3 │COGs shared by and specific to *Spongia officinalis* and *Rhopaloeides odorabile* (A) and *S. officinalis*, *R. odorabile*, *Cymbastela concentrica* and *Cymbastela coralliophila* (B).** Results derive from COG annotations of assembled metagenomes retrieved in this study (*S. officinalis*) and by Fan et al. (2012) (*R. odorabile, C. concentrica and C. coralliophora*).

**References**

Fan, L., Reynolds, D., Liu, M., Stark, M., Kjelleberg, S., Webster, N. S., et al. (2012). Functional equivalence and evolutionary convergence in complex communities of microbial sponge symbionts. *Proc. Natl. Acad. Sci. U.S.A.* 109(27)**,** E1878-E1887. doi: 10.1073/pnas.1203287109

Namiki, T., Hachiya, T., Tanaka, H., and Sakakibara, Y. (2012). MetaVelvet: an extension of Velvet assembler to de novo metagenome assembly from short sequence reads. *Nucleic Acids Res.* 40:e155. doi: 10.1093/nar/gks678
