# Supplementary figures and images for: Comparative Metagenomics Reveals the Distinctive Adaptive Features of the Spongia officinalis Endosymbiotic Consortium
Source: Front Microbiol. 2017 Dec 14;8:2499. doi: 10.3389/fmicb.2017.02499 (PMC5735121; doi:10.3389/fmicb.2017.02499)

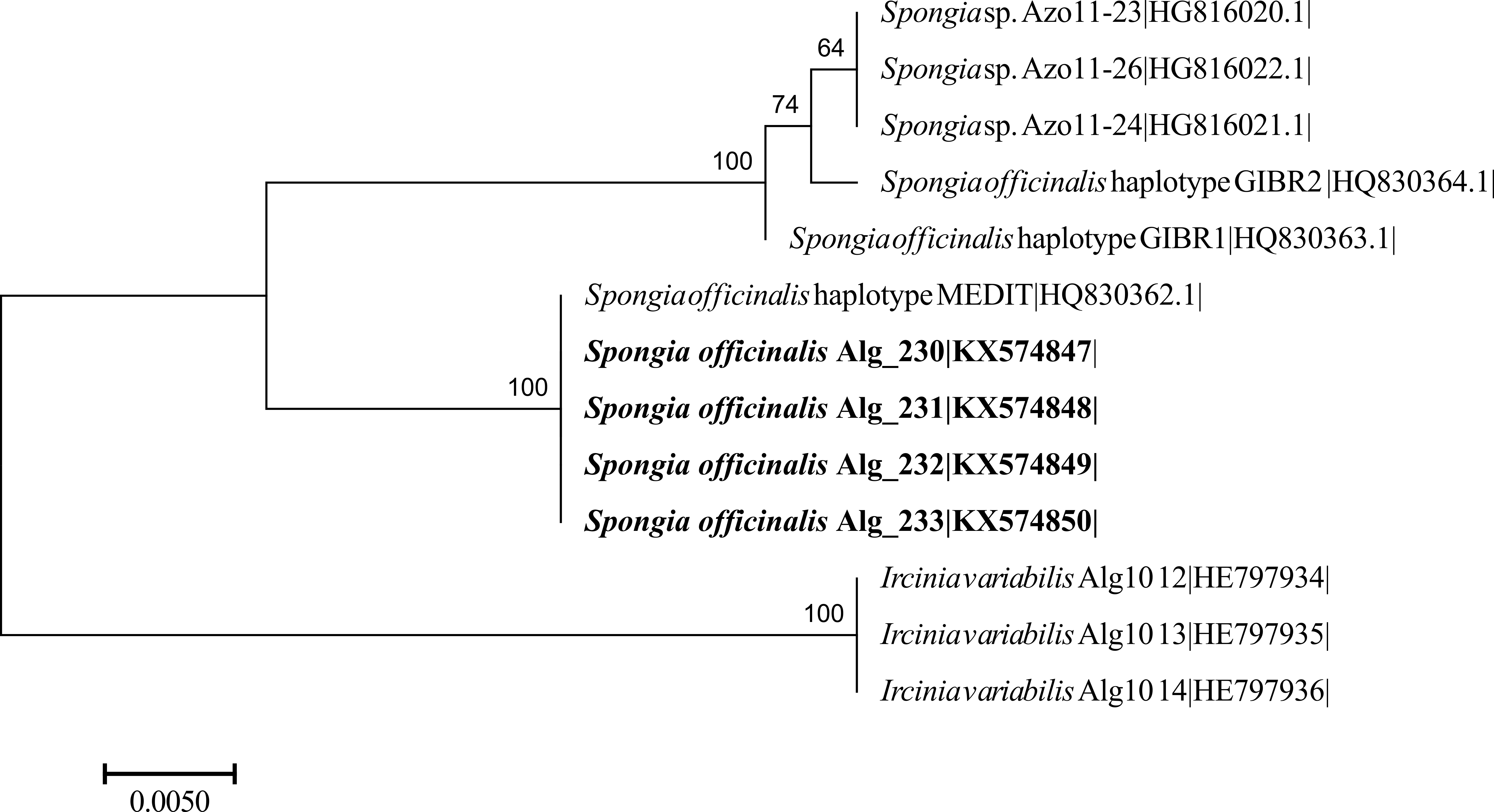

Supplement: Supplementary file 8 [file Image_1.TIF]

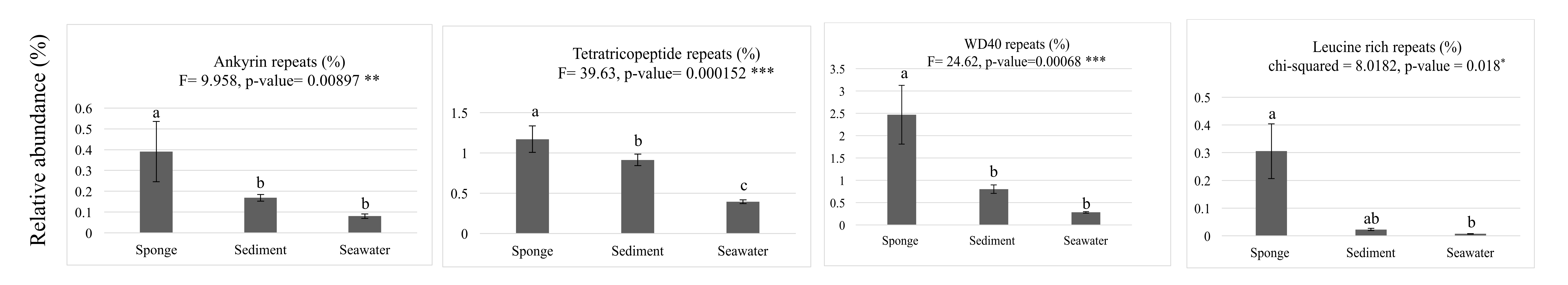

Supplement: Supplementary file 9 [file Image_2.TIF]

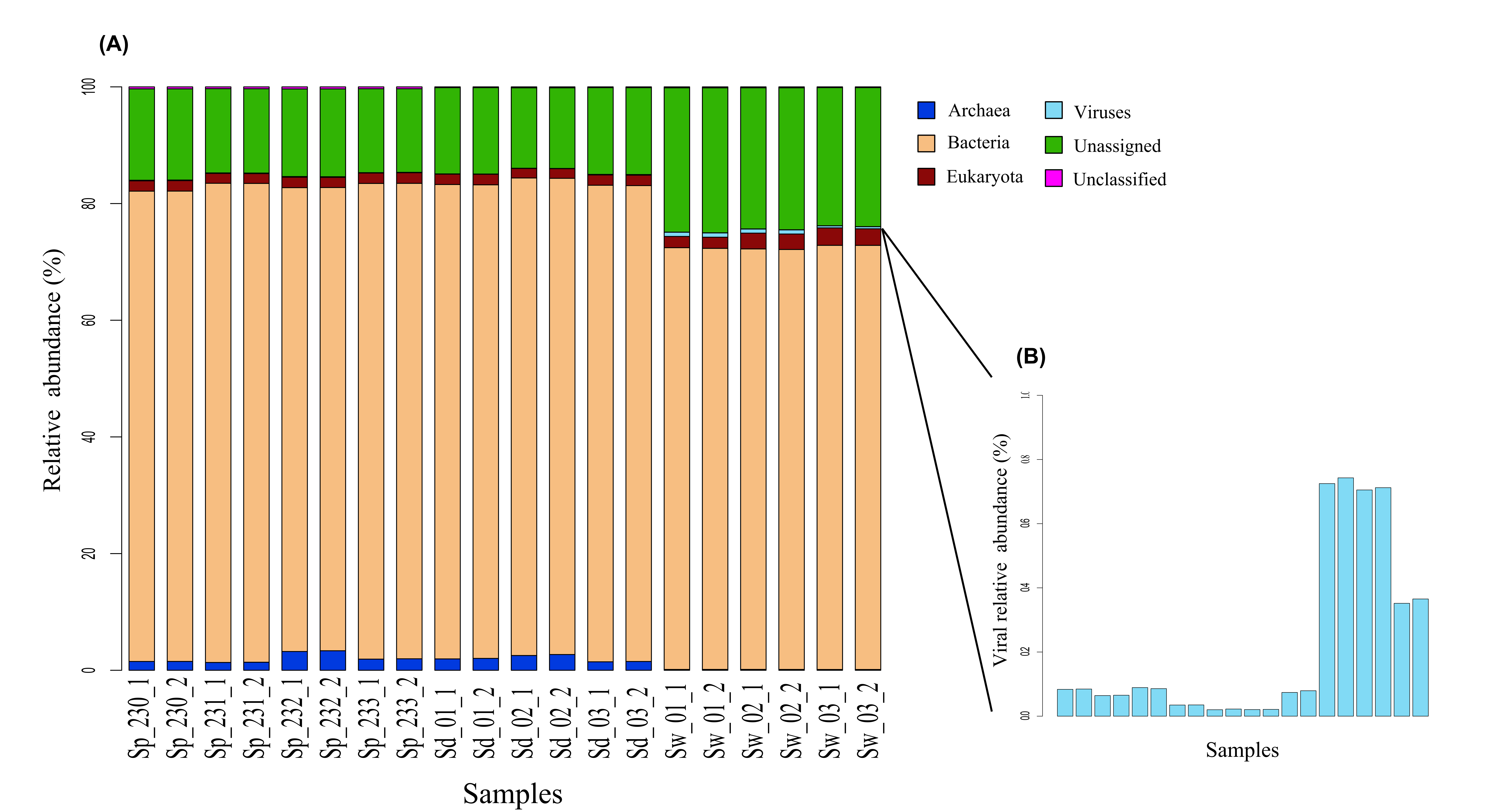

Supplement: Supplementary file 10 [file Image_3.TIF]
